# Fulminant Postoperative Peritonitis Following Cocaine Packet Rupture in a Body Packer

**DOI:** 10.7759/cureus.111475

**Published:** 2026-06-25

**Authors:** Younes Hamdi, Mohamed A Fehdi, Badria Aggoug, Dafir Asmae, Mohammed Mouhaoui

**Affiliations:** 1 Emergency Department Trauma Center, Ibn Rochd University Hospital, Casablanca, MAR; 2 Faculty of Medicine and Pharmacy of Casablanca, Hassan II University, Casablanca, MAR

**Keywords:** body packing, cocaine toxicity, ct imaging, drug smuggling, gastrointestinal foreign bodies, multiorgan dysfunction, packet rupture, ‎peritonitis

## Abstract

Body packing carries a well-documented risk of life-threatening complications, particularly in packet rupture. Postoperative peritonitis following intraperitoneal contamination in the absence of bowel perforation remains an unusual complication. A 32-year-old man was brought to the emergency department by law enforcement following arrest at an international airport on suspicion of drug trafficking. He presented with diffuse abdominal pain and haemodynamic instability consistent with sympathomimetic toxicity. Abdominal computed tomography (CT) confirmed at least 85 cocaine packets throughout the gastrointestinal tract. Rapid clinical deterioration prompted emergency laparotomy, all packets were retrieved, and one was found ruptured, with no bowel perforation or ischaemia. Despite complete surgical decontamination, the patient developed fulminant postoperative peritonitis with disseminated intravascular coagulation and multiorgan failure. He died on postoperative day 2. Intraperitoneal cocaine contamination may lead to a rapidly progressive and potentially fatal course, even after apparently adequate surgical decontamination. Early multidisciplinary management and close postoperative monitoring are essential.

## Introduction

Body packing, defined as the internal concealment of drugs for trafficking, carries a well-documented risk of morbidity and mortality. The illicit substances, most commonly cocaine, are typically packaged in condoms, plastic bags, or other materials and ingested [1]. Packet rupture remains the most feared complication because of the potential for rapid, life-threatening toxicity [2,3].

Management for asymptomatic "body packers" is relatively well-established and generally relies on conservative approaches [4]. Surgery is reserved for failed evacuation or mechanical complications such as bowel obstruction or perforation [5]. The management of packet rupture complicated by systemic toxicity remains challenging, and published data is limited to small case series with few detailed reports of contemporary critical care management [6,7].

Although surgical packet retrieval with peritoneal lavage is the cornerstone of management in symptomatic body packers, the subsequent development of fulminant postoperative peritonitis in the absence of identifiable bowel injury represents an uncommon and poorly characterised complication. Its clinical course and critical care management have not been specifically addressed in the available literature.

We report a fatal case illustrating this uncommon entity and discuss the potential diagnostic and therapeutic dilemmas encountered in the postoperative period.

## Case presentation

A 32-year-old man with no known past medical history was brought to the emergency department by law enforcement following arrest at the international airport on suspicion of drug trafficking. He presented with acute diffuse abdominal pain. No prior medical or surgical history could be elicited, and the patient initially denied any drug ingestion. On admission, he was conscious with a Glasgow Coma Scale (GCS) score of 15/15, but tachycardic at 110 bpm, hypertensive at 150/80 mmHg, tachypnoeic at 28 breaths per minute, and mildly hypoxaemic at 94% on room air, with a temperature of 37.8°C. Abdominal examination revealed distension and diffuse tenderness without peritoneal signs. Bowel sounds were diminished. Electrocardiogram showed sinus tachycardia without ischaemic changes.

Baseline investigations revealed mildly elevated inflammatory and cardiac markers. Renal, hepatic, blood count, and coagulation parameters were unremarkable.

Plain abdominal radiography demonstrated multiple oval radiopaque foreign bodies along the colonic framework, with moderate colonic distension (Figure 1). Computed tomography (CT) confirmed the presence of numerous oval hyperdense foreign bodies in the stomach and colon (Figures 2-3). The packets were similar in size and shape, measuring approximately 47×20 mm, with a mean attenuation of approximately 285 Hounsfield units, consistent with cocaine-containing packages. Discrete fat stranding in the right parietocolic gutter was identified with no free gas, bowel obstruction, or vascular complication.

**Figure 1:**
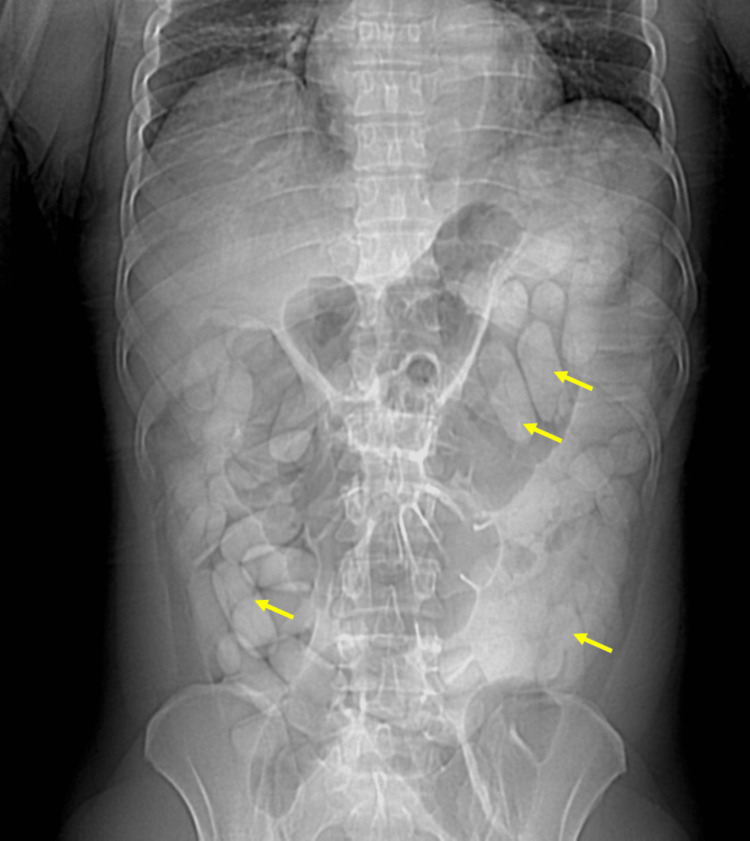
Plain abdominal radiograph showing multiple oval radiopaque foreign bodies distributed along the colon, suggestive of ingested drug packets (arrows)

**Figure 2:**
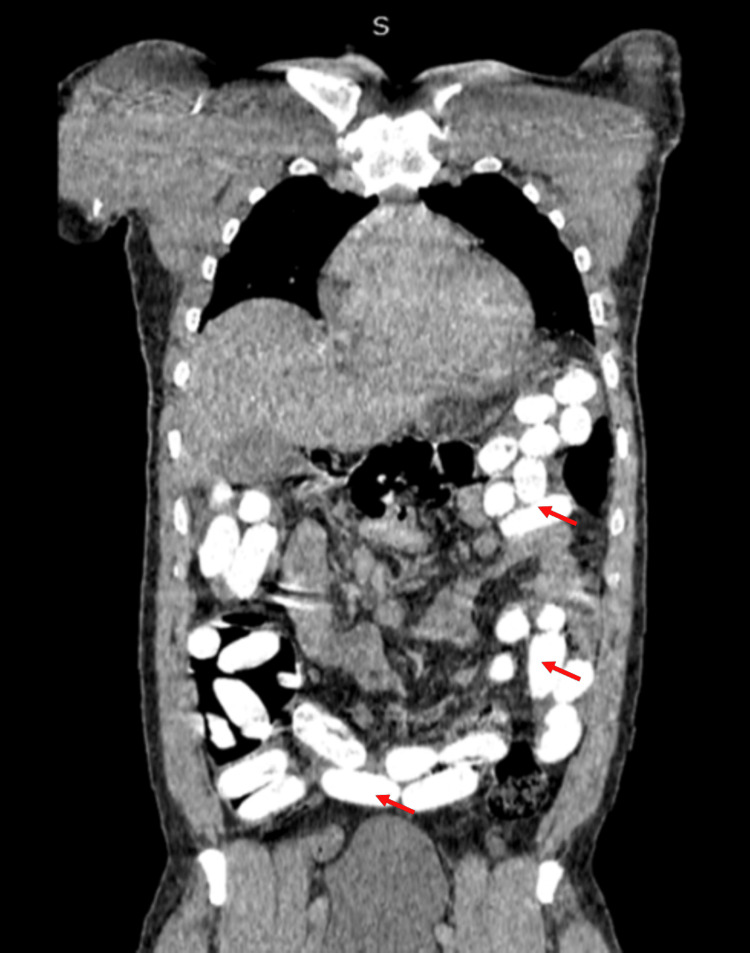
Axial abdominal computed tomography scan showing numerous hyperdense oval packets within the stomach and colon (arrows)

**Figure 3:**
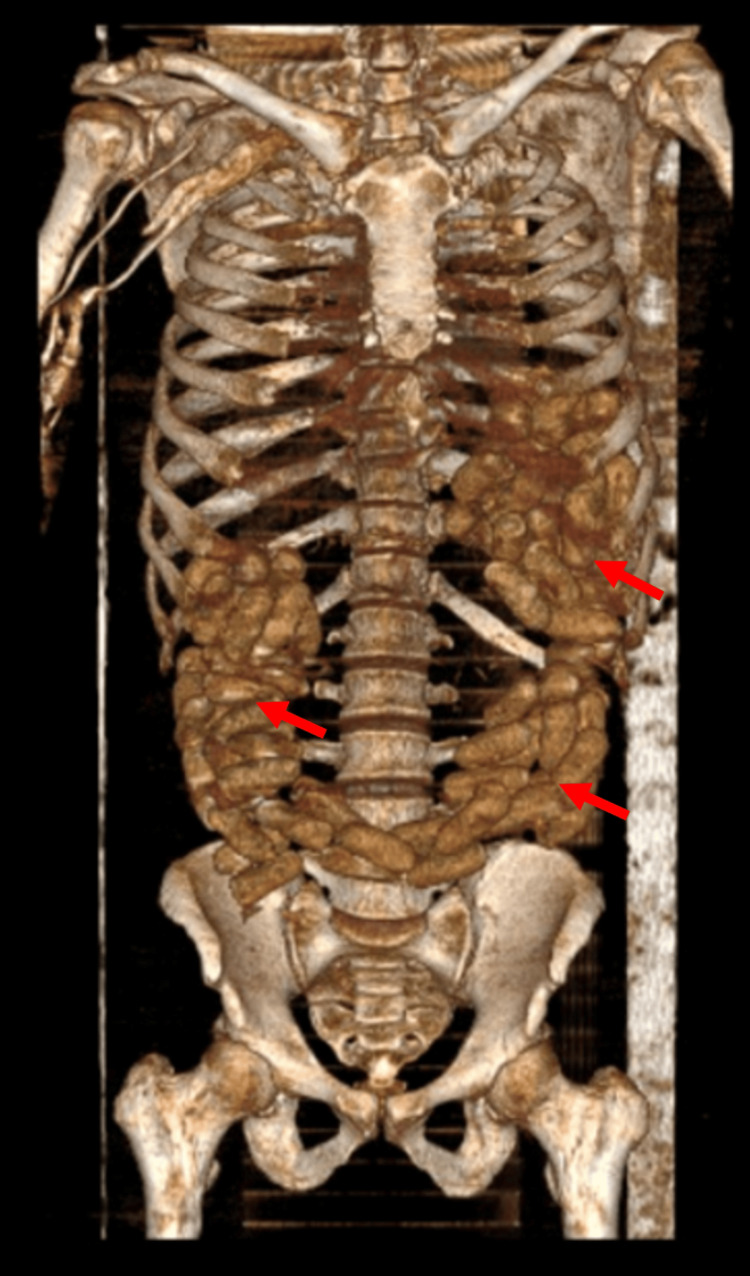
Three-dimensional computed tomography reconstruction showing the distribution of multiple ingested packets (arrows) throughout the gastrointestinal tract

Approximately one hour following admission, the patient developed rapid clinical deterioration. Abdominal pain worsened, GCS score decreased to 12/15, blood pressure rose to 180/100 mmHg, heart rate rose to 130 bpm, and oxygen saturation fell to 90% on 6 L/min supplemental oxygen via facemask, with new non-specific lateral ST-segment changes on repeat electrocardiogram (ECG). This clinical picture was interpreted as acute cocaine toxicity most likely secondary to packet rupture, and emergency surgical intervention was decided without delay. Emergency laparotomy was performed in the presence of law enforcement officers. Intraoperative findings confirmed 85 cocaine packets distributed throughout the gastrointestinal tract (Figure 4). At least one packet was clearly ruptured (Figure 5). All packets were retrieved under manual guidance via sequential enterotomies without the need for bowel resection. Extensive peritoneal lavage was performed with 10 litres of warm isotonic saline until the effluent was macroscopically clear. No bowel perforation, ischaemia, or mesenteric vascular injury was identified at the time of surgery. The total operative time was approximately four hours.

**Figure 4:**
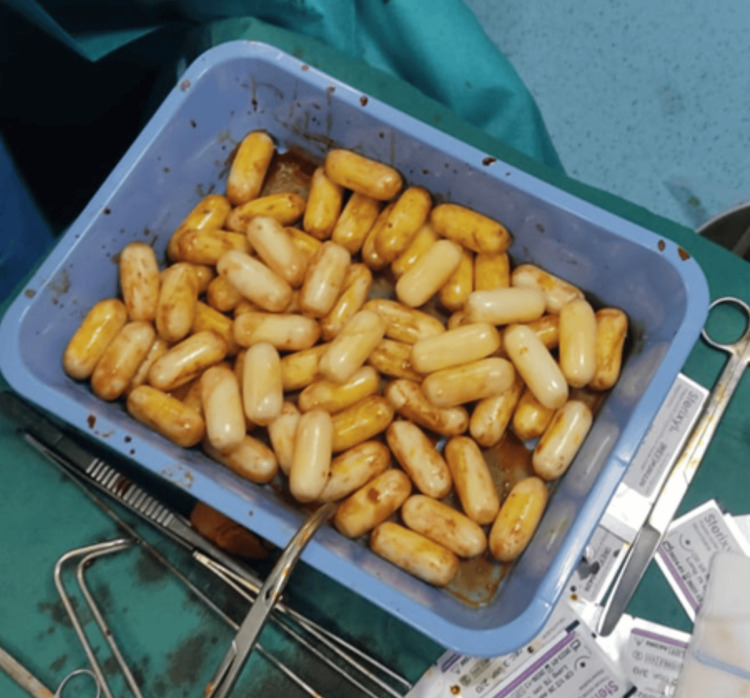
Intraoperative photograph showing the extracted cocaine packets following laparotomy

**Figure 5:**
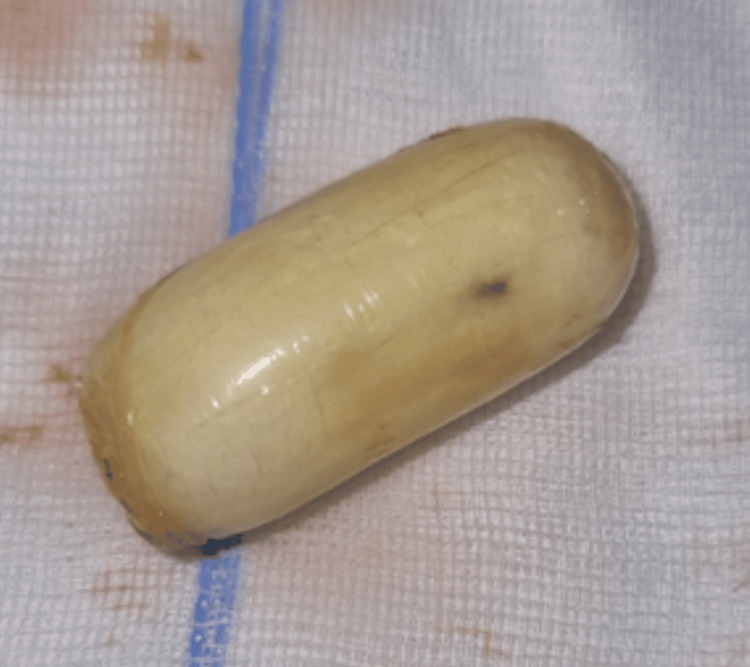
Photograph of a ruptured cocaine packet removed during surgery

The patient was transferred to the surgical intensive care unit and required mechanical ventilation, vasopressor support, continuous benzodiazepine sedation, and broad-spectrum antimicrobial therapy. Over 48 hours, he developed generalised abdominal rigidity, absent bowel sounds, and increased peritoneal fluid on bedside ultrasound, consistent with diffuse peritonitis. Laboratory investigations revealed a markedly elevated inflammatory response, hepatic cytolysis, severe coagulopathy, thrombocytopaenia, hyperlactataemia, and acute kidney injury (Table 1). Surgical re-intervention was deemed unfeasible given refractory vasoplegic shock, severe coagulopathy, and progressive multiorgan failure. The patient died on postoperative day 2.

**Table 1:** Laboratory findings at admission and postoperative day 2 CRP: C-reactive protein; WBC: white blood cell; CK: creatine kinase; AST: aspartate aminotransferase; ALT: alanine aminotransferase; PT: prothrombin time; aPTT: activated partial thromboplastin time

Examinations	On admission	Postoperative day 2	Laboratory reference values
Inflammatory markers			
CRP (mg/L)	12	320	<6
Procalcitonin (µg/L)	-	28	<0.5
WBC (×10⁹/L)	8.75	22	4-10
Cardiac markers			
Troponin I (ng/mL)	0.08	-	<0.04
CK (U/L)	420	-	30-200
Hepatic function			
AST (U/L)	34	151	<50
ALT (U/L)	41	202	<55
Renal function			
Creatinine (mg/dL)	0.80	3.62	0.57-1.10
Lactate (mmol/L)	-	7.8	<2
Coagulogram			
Platelets (×10⁹/L)	322	32	150-450
PT (%)	-	20	70-100
aPTT ratio	-	2.4	<1.2

## Discussion

Cocaine body packing carries significant morbidity and mortality, with outcomes influenced by packet quantity, substance type, and structural integrity [2,3]. Rupture of even a single packet may release a lethal dose [1]. Two clinical syndromes are recognised: a toxic syndrome from systemic absorption and a mechanical syndrome from obstruction or perforation [6]. CT is the gold standard for packet detection and complication assessment, though operative decisions may be driven primarily by clinical deterioration [8].

Asymptomatic patients are managed conservatively, with close observation and whole-bowel irrigation [4,7,9]. Symptomatic patients require intensive care support with benzodiazepines, cardiovascular stabilisation, and surgical removal where indicated [5,6]. In our patient, the decision to operate was driven by rapid clinical deterioration with haemodynamic instability and signs of cocaine toxicity, consistent with current management principles.

Peritonitis in body packing most commonly arises from bowel perforation secondary to packet impaction [1]. Published fatal cases predominantly involve either massive systemic toxicity or mechanical complications such as obstruction or perforation [1,6]. To our knowledge, postoperative peritonitis following surgical management of a packet rupture without identifiable bowel injury has not been previously reported as a distinct entity. In our patient, the absence of bowel perforation, ischaemia, or mesenteric injury was confirmed intraoperatively, and extensive peritoneal lavage was performed until the effluent was macroscopically clear, making a missed surgical complication unlikely, although the persistence of microscopic contamination cannot be entirely excluded.

The mechanism underlying the fatal postoperative course most likely resulted from a combination of ongoing systemic cocaine toxicity and secondary septic peritonitis. The initial rapid deterioration was likely driven by systemic cocaine toxicity, whereas the subsequent refractory shock and inflammatory response may have been amplified by secondary bacterial peritonitis. Multiple sequential enterotomies, although necessary for packet retrieval, may have facilitated bacterial translocation [10]. Systemic cocaine toxicity, through sustained sympathomimetic stimulation and vasoconstriction, likely contributed to multiorgan failure independently of the abdominal source. Whether direct chemical peritoneal irritation played an additional role cannot be excluded, but chemical peritonitis is not a recognised complication of cocaine toxicity in the available literature, and this mechanism remains speculative in the absence of peritoneal fluid analysis, microbiological sampling, and post-mortem examination [11].

This case illustrates the diagnostic and therapeutic challenges that may arise in the postoperative period following body packing surgery. When peritonitis develops in a sedated, mechanically ventilated patient on vasopressor support, classical clinical signs are unreliable, and the diagnosis relies primarily on biological and imaging criteria [12]. In this context, the haemodynamic deterioration may be further confounded by ongoing systemic cocaine toxicity, making early recognition particularly difficult. Relaparotomy for source control, although indicated for postoperative peritonitis, was deemed unfeasible given refractory shock and severe coagulopathy [12]. Based on this case, we suggest that a low threshold for early biological re-evaluation and cross-sectional imaging should be maintained in the postoperative period following body packing surgery, acknowledging that imaging may itself be precluded by haemodynamic instability.

The medicolegal context adds a layer of complexity often underappreciated in clinical reporting. The presence of law enforcement during clinical encounters, patient denial of drug ingestion, and the constraints of a forensic setting may collectively limit history-taking and complicate the therapeutic relationship [13]. In our case, autopsy data remained inaccessible due to ongoing judicial proceedings, precluding definitive conclusions on the underlying cause of death.

## Conclusions

This case adds to the limited literature on a potentially fatal complication of body packing that may unfold despite apparently adequate surgical management. Body packers may present with deceptively mild initial symptoms before rapid deterioration, and a high index of suspicion must be maintained throughout the observation period. Close postoperative vigilance and early multidisciplinary involvement remain essential in this setting.
