# Correction: Fulminant Postoperative Peritonitis Following Cocaine Packet Rupture in a Body Packer

**DOI:** 10.7759/cureus.c459

**Published:** 2026-07-06

**Authors:** Younes Hamdi, Mohamed A Fehdi, Badria Aggoug, Dafir Asmae, Mohammed Mouhaoui

**Affiliations:** 1 Emergency Department Trauma Center, Ibn Rochd University Hospital, Casablanca, MAR; 2 Faculty of Medicine and Pharmacy of Casablanca, Hassan II University, Casablanca, MAR

This article has been corrected to change all authors' affiliations to the following: Emergency Department Trauma Center, Ibn Rochd University Hospital, Casablanca, Morocco, and Faculty of Medicine and Pharmacy of Casablanca, Hassan II University, Casablanca, Morocco.

